# The optimal therapeutic irisin dose intervention in animal model: A systematic review

**DOI:** 10.14202/vetworld.2020.2191-2196

**Published:** 2020-10-20

**Authors:** Foad Alzoughool, Mohammad Borhan Al-Zghoul, Saad Al-Nassan, Lo’ai Alanagreh, Dana Mufleh, Manar Atoum

**Affiliations:** 1 Department of Medical Laboratory Sciences, Faculty of Applied Medical Sciences, The Hashemite University, Zarqa, Jordan; 2 Basic Veterinary Sciences, School of Veterinary Medicine, Jordan University of Science and Technology, Irbid, Jordan; 3 Department of Physical and Occupational Therapy, Faculty of Applied Medical Sciences, The Hashemite University, Zarqa, Jordan

**Keywords:** animal model, Irisin, systematic review, therapeutic dose

## Abstract

**Background and Aim:**

Irisin, a novel myocyte-secreted hormone, was proposed to mediate some of the beneficial effects of exercise such as browning of adipocytes, thermogenesis, and metabolic homeostasis. Recently, several animals’ models’ studies have been performed to investigate the therapeutic impact of irisin in several disorders. Several interventional trials used different doses. However, optimum dose was not determined. This systematic review aims to identify the optimal dose of interventional irisin in mice and rat animal models.

**Materials and Methods:**

Online databases PubMed, Google Scholar, and Springer were systematically searched from 2012 to 2019. The words searched were irisin, irisin and animal model, physical activity, and irisin and irisin dosage. Non-irisin doses, *in vitro* studies, and factors influencing irisin levels were excluded.

**Results:**

Eleven of the total 391 qualifying studies were included. A daily injection of 500 μg/kg irisin may be the optimum dose of effect in mice and rats.

**Conclusion:**

More studies are required to determine the optimum dose of irisin to be used as a therapeutic intervention based on animal model.

## Introduction

Recently, muscles were recognized as an endocrine organ releasing myokines [1]. Myokines are important in regulating multiphysiological and metabolic pathways to induce metabolism through communication with several tissues such as pancreas, liver, and many more [2,3]. Irisin is a novel myokine hormone secreted by myocytes that have been suggested to mediate some of the beneficial effects of exercise such as, prevents weight gain and metabolic dysfunction [1]. This muscle-derived myokine released from the fibronectin type III domain containing 5 (FNDC5) after its extracellular portion cleavage of and secreted to the circulation. FNDC5 was proposed to induce browning of subcutaneous adipocytes and thermogenesis by increasing the levels of the uncoupling protein 1 (UCP1) in both the *in vitro* and the *in vivo* mouse models [1].

It is hypothesized that FNDC5 induces the differentiation of a subset of white adipocytes into brown adipocytes and mediates the beneficial effects of exercise on metabolic homeostasis and energy expenditure [4]. Since the exercise is an excellent therapeutic intervention for pathologies such as obesity, type 2 diabetes, cardiovascular, and neurodegeneration, irisin has been considered a potential therapeutic candidate to replicate the physiological effects of exercise and thus treat several diseases [5]. It was demonstrated that on exercise stimulation, and through the peroxisome proliferator-activated receptor gamma coactivator 1-alpha, the expression of FNDC5 in muscle is enhanced and irisin is secreted, inducing the activation of thermogenesis genes in certain adipocytes [6]. Irisin was reported to be detected in human and mouse plasma and increased approximately 1.5-fold after exercise compared with control, using Western blotting [7]. Irisin has 100% identical sequence in mouse, rat, and humans. Irisin as a hormone is predicted to have therapeutic applications related to different forms of diseases such as obesity, cancer, bone dysfunction, and muscle atrophy [8].

The study aimed to determine the optimal dose of interventional irisin in animal model studies to help future experimental studies in decreasing the irisin consumption with a cost-effective manner.

## Materials and Methods

### Ethical approval

All procedures of this study were approved by the Animal Care and Use Committee (JUST-ACUC) of Jordan University of Science and Technology.

### Methods

**Figure-1:**
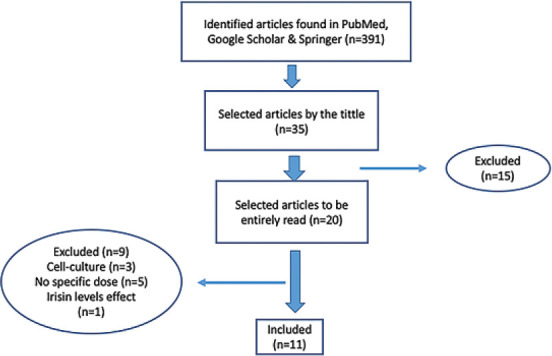
Inclusion criteria of systematic review.

A detailed systemic literature search was performed in international online databases: Science Direct and Scopus; to determine the optimal dose of irisin in mice and rat animal models. We started looking for irisin in general to understand the mechanism of this myokine, then we had searched the irisin and physical activity abstracts, especially in animal models (rats and mice). All abstracts selected have been from 2012 up to 2019. In detail, we identified studies that were selected by searching for the following keywords: Irisin doses in the animal model, physical activity related to irisin, therapeutic irisin effect on rats and mice, and the myokine irisin. We found 391 studies the best match default sort order in the PubMed and 336 studies in Google Scholar and Springer for the most recent. We excluded articles with non-irisin doses, *in vitro* studies, and factors that affect irisin level, as shown in Figure-1.

### Study selection

Studies providing data on different therapeutic doses and routes of irisin, *in vivo*, and well-defined studies identified all diseases that therapeutically require irisin were included in the study. *In vitro* and investigational studies that explain factors affected by irisin levels were excluded from the study. In addition, only dose-dependent irisin studies that provided suitable data about the therapeutic dose of irisin were included in the study. Table-1 shows the criteria for inclusion and exclusion for the studies selection.

**Table 1:** Inclusion and exclusion criteria of systematic review.

Inclusion criteria
All included studies in this systematic review depend on the following:
Effect of irisin in rats and mice animal models.
Physical activity-related irisin.
Therapeutic irisin for specific disorders.
Irisin dose on FNDC5 gene expression. Studies from 2012 until the present.

**Exclusion criteria**

All excluded studies in this systematic review depend on the following:
*In vitro* studies
Non-dose mentioned studies.
Any dose-effect study on an animal model other than mice or rats.
Irisin association studies with certain factors or diseases.
Not a well-defined dose such as; “weekly or daily doses.”

### Data extraction

The following data were extracted and presented in Table-2: Author(s), year, (dose, time, and unit), mice and rat type, company of irisin production, and aims of the study. Data were obtained independently of 11 studies using a standardized criterion. All the selected studies provided detailed information for the requested data.

**Table 2:** Included studies summarization.

Reference	Type of injection	Aims	Company produced irisin	Mice type and info	Significant dose μg/ kg	Dose frequency
Asadi *et al*. [16]	ICV Intracere broventricularly	Protect against brain ischemic injury	Orb180476 Biorbyt Cambridge, UK	Male Swiss albino mice 30-35 g 42 mice	7.5 μg/kg	Single dose after ischemia
Colaianni *et al*. [9]	Injected only (not mentioned)	Low=Increase in cortical bone and strength High=Cause browning of adipose tissue	AG-40B-0103 Adipogen	C57BL/6 male 2 months	Low dose 100 μg/kg High dose 500 μg/kg	Weekly (4 weeks) Daily (3500 μg/kg weekly) for 2 weeks
Zhao *et al*. [17]	Tail intravenous injection	Beneficial against ischemia-reperfusion injury after perforator flap grafting in rats	NM	Sprague-Dawley rats 200-250 g 48 n	2 μg/kg	Daily The effect was significant on day 7
Dameni *et al*. [19]	Intrathecal administration	Pain threshold and expression rate of GABAB receptors in peripheral neuropathic pain model	Orb 180477 Biorbyt Cambridge, UK	Male Wistar rats 200-250 g	1 μg/kg	In one dose, it was affected on pain threshold but daily for 2 weeks have no effect.
Duan *et al*. [13]	Intraperitoneal injection	Could lower the glucose in diabetic mice	Gene expression in yeast *P. pastoris* GS115	C57BL/6J male 3 weeks 10-12 g 54 n	1 mg/kg	Daily for 3 weeks
Liu *et al*. [15]	Intraperitoneal injection	Low-dose irisin treatment alleviated cardiac fibrosis and left ventricular function in the diabetic mice	Gene expression in yeast *P. pastoris* pPICZaA plasmid Life Technology	C57BL/6J male 25-30 g	500 μg/kg	Daily for 16 weeks
Colaianni *et al*. [10]	Intraperitoneal injection	Prevents and restores bone loss and muscle atrophy	Adipogen International (San Diego, USA)	C57BL/6 male 2 months 64 n	100 μg/kg	Weekly for 4 weeks (once a week)
Zhu *et al*. [12]	Intraperitoneal injection	Irisin Increased the number and improved the function of endothelial progenitor cells in diabetes mellitus mice	Phoenix Pharmaceuticals	C57BL/6 mice (4 weeks old; 13-15 g	500 μg/kg	Daily for 8 weeks
Majeed *et al*. [18]	Intraperitoneal injection	On body mass index, serum insulin, luteinizing hormone, and testosterone levels in obese female	CUSABIO	BALB/c female 6-8 week 25±5 g	200 μg/kg	Daily for 7 days
Shao *et al*. [14]	Intraperitoneal injection	Effects of irisin on LPS-induced acute lung injury in mice	Phoenix Pharmaceuticals Inc. (Karlsruhe, Germany)	C57BL/6J male 6-8 week	500 μg/kg	Daily for 21 days
Zhang *et al*. [11]	Intraperitoneal injection	Irisin can potentially prevent obesity and associated type 2 diabetes by stimulating expression of WAT browning-specific genes	Gene expression in yeast *P. pastoris* pPICZaA plasmid life	C57BL/6 mice 10 weeks old feed 35-43 g 16 n	500 μg/kg	Daily for 14 days

P. pastoris=Pichia pastoris

## Results

### Study selection

The literature review method is outlined and illustrated as a flowchart in Figure-1. In the final phase, 11 qualifying studies were included in this review and evaluated for inclusion and exclusion criteria. Table-1 outlines the inclusion/exclusion criteria.

### Study characteristics

Overall, 391 studies were included in this systematic review. All included studies were published between 2012 and 2019, as irisin was discovered in 2012. The included studies were carried out on male and female rats and mice. Seven studies were conducted using male C57BL/6 male [9-15], four of the studies used different types of rats and mice which are Swiss albino mice, Sprague-Dawley rats, Wistar rats, and BALB/c female [16-19]. Only one study described the exposure to a single dose of irisin [16] while other studies described the exposure of either a daily or weekly dose. The dosage and duration of exposure differed between studies from either 4 weeks (once a week) or daily for a given time (Table-2). All studies reported positive effects on the use of irisin as a therapeutic drug, some showing a shift in gene expression of FDNC5 and UCP-1. Irisin used in these experiments was obtained from different production companies; Orb 180476 Biorbyt Cambridge, UK [7,20], gene expression in yeast *Pichia pastoris* GS115 [13], gene expression in yeast *P. pastoris* pPICZaA plasmid Life Technology [11,15], Phoenix Pharmaceuticals [12,14], AG-40B-0103 Adipogen [9,10], and CUSABIO [18]. One study did not mention the company that manufactured irisin [17]. Most studies used intraperitoneal injection except for Dameni *et al*. [19] that used intrathecal administration [17], used an intravenous tail injection and [16] ICV intracerebroventricularly. One study did not mention the route of administration for irisin [9]. All the included studies are summarized in Table-2.

## Discussion

This systematic review is aimed to clarify the doses of irisin on the animal models to determine the optimum dose, as there are inconstancies in irisin dose in the previous studies. These following paragraphs describe the doses of irisin and different animals’ diseases models.

### Irisin doses affecting brain ischemia

Two studies in this review showed a significant effect of irisin dose on brain ischemia. The first study one indicated significant downregulation of Bax and caspase-3 gene expressions [16]. However, irisin showed a protective effect against some brain disorders such as stroke damage [16]. The second study investigated the irisin protective effect against ischemia perfusion [17]; irisin was reported to prevent endothelial damage from body oxidant. Furthermore, this study showed that irisin indirectly affects other signaling pathways, such as; PI3K/Akt. No optimal dose for both studies was reported, in Asadi *et al*. [16] study, a single dose of 7.5 μg/kg after ischemia, while Zhao *et al*. [17] used a daily dose of 2 μg/kg where it was a time-dependent dose and the effect was significant on day 7.

### Irisin doses affecting cortical bone mass and bone loss

Several evidences supporting the biological association of irisin between skeletal muscle and bones. In 2015, Colaianni *et al*. [9] reported that injecting mice with 100 μg/kg of recombinant irisin for 1 week caused an increase in strength and mass of cortical bone, whereas injecting mice with a higher dose of irisin (3500 μg/kg/week) induced browning of adipose tissue. At the molecular level, a low dose of irisin improved the gene expression level of Opn and Sost genes expressed in skeletal muscle with no effects on UCP1 gene expression level that is expressed in white adipose tissue. However, irisin led to upregulation of the gene expression of Atf4, Runx2, Osx, Lrp5, β-catenin, Alp, and Col1a1 gene that promotes osteogenesis. These findings presented a piece of supporting evidence that irisin may be a possible candidate for the link between muscles and osteoblasts, as irisin may help forming new bone. In another study for the same team, injected mice with 100 μg/kg weekly for 4 weeks (once a week) prevented and restored bone loss and muscle atrophy, which provides further proof of the role of irisin in both physically immobile and osteoporotic elderly patients [10].

### Irisin doses related to diabetes, obesity, serum insulin, and body mass

Exercise is an excellent therapeutic intervention for pathologies such as obesity, type 2 diabetes, cardiovascular, and neurodegeneration; irisin has been considered a potential therapeutic candidate to replicate the physiological effects of exercise and hence treat many diseases [5]. An intriguing finding by injecting mice with 500 μg/kg irisin daily for 2 weeks reduced the body weight and improved glucose homeostasis through increasing the expression of UCP-1 gene which helped in browning white adipose tissue, this could be a preventive countermeasure for obesity and associated type 2 diabetes [11]. 1.0 mg/kg irisin was the ideal dose of daily intraperitoneal injection for 3 weeks that significantly decreased the glucose in diabetic mice [13]. In another study, irisin significantly reduced the elevated BMI, serum insulin, and LH levels in female BALB/c mice injected with 200 μg/kg irisin daily for 1 week [18]. This supports the hypothesis that irisin is an effective treatment for metabolic disorders [18].

### Irisin doses related to diabetic cardiomyopathy

Intraperitoneal injection of 500 μg/kg irisin in mice daily for 16 weeks alleviated cardiac fibrosis by inhibiting endothelial-to-mesenchymal transition by enhancing the expression of MMP which plays an important part in the abnormal of ECM synthesis and/or degeneration [15]. On the other hand, 1500 μg/kg irisin in mice daily for 16 weeks disrupted normal MMP expression and induced proliferation and migration of cardiac fibroblast, resulting in excess collagen deposition, indicating bidirectional effect on diabetic cardiomyopathy by dose-dependent model [15].

### Irisin dose related to acute lung injury (ALI)

Interleukin (IL)-1β and tumor necrosis factor (TNF)-α and other cytokines such as MCP-1 and IL-6 are considered to be elevated during pulmonary inflammation [21]. The potential effect of irisin as an anti-inflammatory for LPS induced the ALI was investigated by intraperitoneal injection of mice by 500 μg/kg irisin daily for 3 weeks, the results showed that irisin suppressed the production of IL-1β, IL-6, MCP-1, and TNF-α supporting the anti-inflammatory action of irisin and giving evidence that irisin may be a potential therapy for the treatment of pulmonary inflammation [14].

### Irisin effects on endothelial progenitor cells (EPCs)

It is well known that endothelial repair and neovascularization are induced by EPCs [22]. However, the reduced number and function of EPCs are predominant in patients with diabetes mellitus [20]. Intraperitoneal injection of 500 μg/kg irisin daily for 8 weeks increased the number and improved the function of EPCs in diabetes mellitus mice by influencing the PI3K/Akt/eNOS pathway, indicating irisin as a potential interventional therapy to improve EPC function for diabetic patients [12].

### Irisin related to the pain threshold

Physical activities were reported to reduce acute pain in healthy individuals [23]. A study reported that a single intrathecal administration of 1 μg/kg irisin increases the pain threshold while repeating the dose for 2 weeks mimicking the chronic injection but its chronic injection does not have an effect on pain reduction [19].

Although the therapeutic effect on animal models is minimal, the *in vitro* studies and the association of plasma irisin and the gene expression with many conditions are studied broadly. The effect of irisin on organs, muscle, adipose tissue, or heart had been investigated in limited studies [24,25]. FNDC5 gene and irisin protein are expressed in monkeys’ hypothalamic-pituitary-gonadal axis [25]. Besides, irisin showed a novel factorial involvement in the regulation of spermatogonial activities in the testes of primates [25]. Moreover, irisin showed the ability to inhibit pathological cardiac hypertrophy *in vitro* [26].

All studies included in our systematic review presented proof of the effectiveness of irisin intervention as a therapeutic agent for various conditions, in addition to mimicking individuals’ physical activity benefits. Irisin intervention reported preventing brain ischemia and ischemic reperfusion injury, preventing and restoration of bone loss, and preventing of muscle atrophy. Irisin also showed a promising therapy for metabolic disorders, induced cardiac fibroblast proliferation and migration in diabetic mice, improved EPCs function for diabetic patients, provided a potential therapy for the treatment of pulmonary inflammation, and increased the pain threshold leading to pain reduction. Although there is no optimal irisin dose that showed a therapeutic effect, and not all studies used the same type of mice, a daily injection of 500 μg/kg irisin might be considered the optimum dose of effect in C57BL/6 mice. As shown in Table-1, five out of seven studies used the dose of 500 μg/kg daily. The sixth study used daily 1000 dose μg/kg in C57BL/6 mice, while the seventh one used only 100 μg/kg daily to protect against bone loss. The finding concluded that the optimum dose of irisin in C57BL/6 mice was 500 μg/kg daily. In male Swiss albino mice, the optimum dose was 7.5 μg/kg to protect against brain ischemia, while the optimum dose in the BALB/c female mice was 200 μg/kg, this difference in the dose could be by type and sex of the mice. In rat model, 1 μg/kg dose was the optimum in male Wistar rats, and 2 μg/kg dose was the optimum in Sprague-Dawley rats. These two doses are similar to each other, so the optimum doses in the rat model might be 1-2 μg/kg.

## Conclusion

No study could be found about the toxicity and safety of the irisin intervention in any different animal models. However, in this review, we summarize all the potential irisin interventions doses used in mice and rats’ animal models between 2012 and 2019, the ideal dose of irisin in mice, in particular, C57BL/6 mice is 500 μg/kg daily, while in rat model is 1-2 μg/kg. More studies are required to evaluate the appropriate dosage of irisin for therapeutic intervention.

## Authors’ Contributions

FA and MBA collected and analyzed the data, formal analysis, funding acquisition, and original draft preparation. SA, LA, MA, and DM helped in designing the methodology and data curation. FA, MBA, LA, and MA reviewed and edited the manuscript. All the authors read and approved the final and revised copy of the manuscript.
